# Analysis of the Coding and Non-Coding RNA Transcriptomes in Response to Bell Pepper Chilling

**DOI:** 10.3390/ijms19072001

**Published:** 2018-07-09

**Authors:** Jinhua Zuo, Yunxiang Wang, Benzhong Zhu, Yunbo Luo, Qing Wang, Lipu Gao

**Affiliations:** 1 Key Laboratory of Vegetable Postharvest Processing, Ministry of Agriculture, Beijing Vegetable Research Center, Beijing Academy of Agriculture and Forestry Sciences, Beijing 100097, China; 2 Beijing Key Laboratory of Fruits and Vegetable Storage and Processing, Beijing Vegetable Research Center, Beijing Academy of Agriculture and Forestry Sciences, Beijing 100097, China; 3 Key Laboratory of Biology and Genetic Improvement of Horticultural Crops (North China) of Ministry of Agriculture, Beijing Vegetable Research Center, Beijing Academy of Agriculture and Forestry Sciences, Beijing 100097, China; 4 Key Laboratory of Urban Agriculture (North) of Ministry of Agriculture, Beijing Vegetable Research Center, Beijing Academy of Agriculture and Forestry Sciences, Beijing 100097, China; 5 Boyce Thompson Institute for Plant Research, Cornell University Campus, Ithaca, NY 14853, USA; 6 Beijing Academy of Forestry and Pomology Sciences, Beijing Academy of Agriculture and Forestry Sciences, Beijing 100093, China; yunxiangjkkl@126.com; 7 Laboratory of Postharvest Molecular Biology of Fruits and vegetables, Department of Food Biotechnology, College of Food Science and Nutritional Engineering, China Agricultural University, Beijing 100083, China; zbz@cau.edu.cn (B.Z.); lyb@cau.edu.cn (Y.L.)

**Keywords:** analysis, non-coding RNA, transcriptomes, bell pepper, chilling injury

## Abstract

Increasing evidence suggests that long non-coding RNAs (lncRNAs), circular RNAs (circRNAs), and microRNAs (miRNAs) have roles during biotic and abiotic stress, though their exact contributions remain unclear. To explore their biological functions in response to chilling in bell pepper, we examined their accumulation profiles by deep sequencing and identified 380 lncRNAs, 36 circRNAs, 18 miRNAs, and 4128 differentially expressed mRNAs in the chilled versus the non-chilled fruit. Gene ontology (GO) and Kyoto Encyclopedia of Genes and Genomes (KEGG) analyses revealed differentially expressed genes and putative ncRNA targets, including transcription factors of multiple classes, such as myeloblastosis (MYB), basic helix-loop-helix (bHLH), and ethylene response factor (ERF) transcription factors (TFs), enzymes involved in bio-oxidation and oxidative phosphorylation (serine/threonine-protein kinase, polyphenol oxidase, catalase, peroxidase, lipoxygenase, and ATPase), and cell wall metabolism-related enzymes (beta-galactosidase, pectate lyase, pectinesterase, and polygalacturonase). On the basis of the accumulation profiles, a network of putatively interacting RNAs associated with bell pepper chilling was developed, which pointed to ncRNAs that could provide the foundation for further developing a more refined understanding of the molecular response to chilling injury.

## 1. Introduction

Bell pepper (*Capsicum annuum*) is important from both nutritional and commercial standpoints because of its high vitamin C content and its widespread production throughout tropical, sub-tropical, and temperate regions [1,2,3]. To maintain the fruit quality, the pepper fruit must be cooled as quickly as possible after harvest [4]. However, pepper fruits are highly sensitive to cold and susceptible to chilling injury (CI) when transported or stored below 7 °C [5]. The main symptoms of chilling injury damage include deterioration of the calyx, sunken lesions, seed browning, and surface pitting [6,7]. CI limits the storage life and leads to a significant degradation of the postharvest nutritional quality and product value. However, cold storage is generally the most effective technology to maintain the quality of postharvest horticultural crops. Thus, it is important to overcome the chilling stress in commercially important chilling-sensitive crops [5,8].

With the development of deep-sequencing technology, numerous non-coding RNAs (ncRNAs) have been discovered in recent years [9]. The ncRNAs can be classified according to their length and function [10,11]. For instance, small ncRNAs of 20–30 nt are mostly microRNAs (miRNAs) and small interfering RNAs (siRNAs), usually associated with transcriptional and translational effects [12]. Medium ncRNAs of 50–200 nt and long ncRNAs (lncRNAs) over 200 nt are associated with splicing, gene inactivation, and translation [13,14]. Unlike linear mRNAs, circRNAs form covalently closed loop structures which originate from tRNAs, exons, introns, or combinations of these molecules to form stable circular RNAs [15,16,17,18,19,20,21]. Recently, both lncRNAs and circRNAs have been suggested to have properties as “miRNA sponges”, whereby they contribute to the regulation of gene expression by operating as competing RNA (ceRNA), influencing a number of distinct biological processes [22,23].

Previously, in a study focused on pepper miRNAs, a comprehensive bioinformatics analysis revealed 11 miRNAs and 54 putative target genes [24]. Via later deep sequencing, 59 known miRNAs and 310 novel miRNAs were found in hot and black pepper [25,26]. The targets of the miRNAs were analyzed and, in some cases, identified as factors associated with fruit development, quality, and stress response [26,27]. In another study, using strand-specific RNA-sequencing, 2505 putative lncRNAs were identified, and many were associated with functions involved in fruit development and quality in hot pepper [28]. To better understand the molecular mechanisms involved in preventing CI, transcriptome profiling analyses of peppers treated with methyl jasmonate (MeJA) and Brassinosteroids (BRs) were performed [5,29]. However, little effort has been focused on the regulation of miRNAs, circRNAs, and lncRNAs in conjunction with mRNA expression during bell pepper chilling, and, as such, the broader non-coding RNA network involved in chilling response remains unclear. 

In this study, high-throughput sequencing was employed to explore the regulation of ncRNAs during bell pepper chilling. We identified 380 lncRNAs, 36 circRNAs, 18 miRNAs, and 4128 differentially expressed mRNAs in response to chilling in pepper fruit. In addition, gene ontology (GO) and Kyoto encyclopedia of genes and genomes (KEGG) analyses revealed that several ncRNAs were involved in the chilling response, such as the WRKY and bHLH transcription factors, key enzymes, including polyphenol oxidase, catalase, peroxidase, and lipoxygenase involved in redox reaction, and cell wall metabolism-related enzymes, such as beta-galactosidase, pectate lyase, and polygalacturonase. Furthermore, the competing endogenous RNAs (ceRNAs) network of lncRNAs, circRNAs, mRNAs, and miRNAs was assessed by examining gene annotation to uncover influenced pathways and processes.

## 2. Results

### 2.1. Identification of Differential Expressed (DE) and Novel Non-Coding RNAs (ncRNAs)

**Figure 1:**
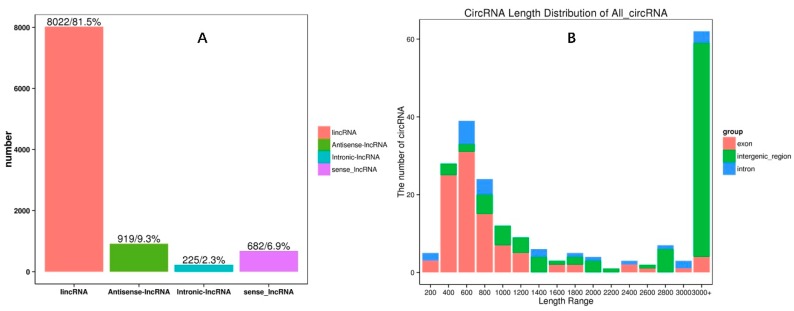
The four kinds of long non-coding RNAs (lncRNAs) were: long intergenic noncoding RNAs (lincRNAs) (8022, 81.5%), antisense-lncRNAs (919, 9.3%), sense lncRNAs (682, 6.9%), and intronic-lncRNAs (225, 2.3%) (**A**).

In our results, 9848 lncRNAs were found: 84 were known lncRNAs, and 9764 were novel lncRNAs found in the control and chilling samples (Table S1). Among them, most of the lncRNAs were lincRNAs (8022, 81.5%), followed by antisense-lncRNAs (919, 9.3%), sense lncRNAs (682, 6.9%), and intronic-lncRNAs (225, 2.3%) (Figure 1A). In addition, 213 novel circRNAs were found, with many emanating from chromosome 8 (Table S1). The majority of circRNAs were over 3000 nt and from intergenic regions, while additional circRNAs were between 400 to 800 nt and derived from exons (Figure 1B, Table S1). In total, 281 miRNAs were found in our libraries with 120 known and 161 novel miRNAs. Most of the novel miRNAs were between 21 and 24 nt. The miRNAs nucleotide bias was also analyzed in our results, and, intriguingly, we found that the first nucleic acid bases were U and A, while the last was G (Table S1). 

**Figure 2:**
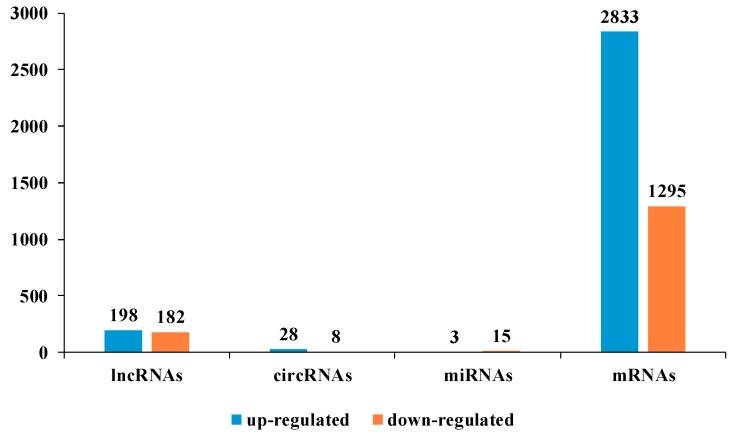
Differentially expressed (DE) ncRNAs: 380 lncRNAs, 36 circRNAs, 18 miRNAs, and 4128 mRNAs were found differentially expressed between the control and chilling groups.

We compared the expression profiles of lncRNAs, circRNAs, miRNAs, and mRNAs between the control and chilling groups, and found that 380 lncRNAs, 36 circRNAs, 18 miRNAs, and 4128 mRNAs were differentially expressed (Figure 2, Table S2). Among them, 198 lncRNAs, 28 circRNAs, 3 miRNAs, and 2833 mRNAs were upregulated, whereas 182 lncRNAs, 8 circRNAs, 15 miRNAs, and 1295 mRNAs were downregulated in the chilling sample compared with the control. The differentially expressed non-coding RNAs are listed in Supplementary Table S2. The differentially expressed lncRNAs and mRNAs were widely distributed on the autosomal chromosomes, while the differentially expressed circRNAs were not found in chromosomes 5, 9, and 11, and their number was the largest in chromosome 1. 

### 2.2. GO and KEGG Pathway Analyses of ncRNAs

**Figure 3:**
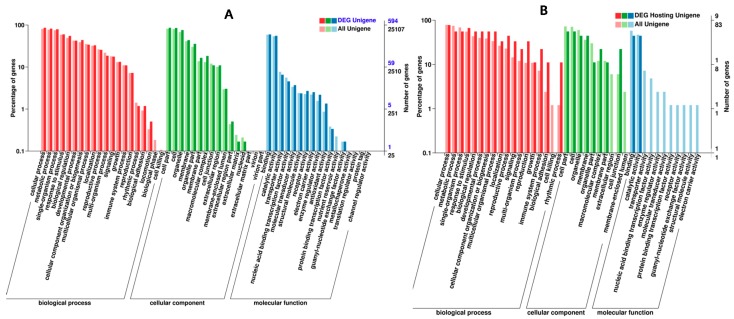
The gene ontology (GO) and Kyoto encyclopedia of genes and genomes (KEGG) analysis of the ncRNAs targets indicated that several important enzymes were involved in the chilling injury (CI).

To explore the potential functions of the differential expressed non-coding RNAs, we performed GO and KEGG analyses. To our knowledge, the lncRNAs could regulate the expression of neighboring and overlapping coding genes; hence, lncRNAs likely regulate related mRNA genes [30]. The cis and trans targets of the differentially expressed lncRNAs were both analyzed, and the most relevant GO terms associated with biological processes and molecular functions contained many important response regulators and key enzymes involved in chilling injury. (Figure 3A, Table S3). The KEGG analysis results showed that the most frequently predicted pathways were involved in oxidative phosphorylation, carbon metabolism, ubiquitin-mediated proteolysis for the *cis*-acting targets of lncRNAs; in contrast, the trans-acting targets of lncRNAs were mainly involved in glyoxylate and dicarboxylate metabolism, carbon fixation in photosynthetic organisms, and carbon metabolism, indicating their specific regulation functions in chilling injury in bell pepper (Tables S3 and S4). 

The function of the differentially expressed miRNAs was also parsed. The most enriched GO terms were related to biological processes, such as response to oxygen-containing compound, response to abiotic stimulus, signal transduction, and hormone-mediated signaling pathway. However, the most relevant GO terms associated with molecular functions were protein binding, protein kinase activity, signaling receptor activity, and ATPase activity (Table S3). KEGG pathway analysis suggested that mRNAs were remarkably enriched in the pathways involved in RNA degradation and transport, peroxisome, mRNA surveillance pathway, plant hormone signal transduction, phenylpropanoid biosynthesis, and folate biosynthesis (Table S4).

So far, the majority of circRNAs has not been functionally annotated [31]. To explore the potential function of differentially expressed circRNAs, GO and KEGG pathway analyses of circRNAs were performed. Our data showed that the most relevant GO terms associated with biological processes were response to temperature stimulus, response to stress, signaling, cell communication, regulation of cellular process, regulation of primary metabolic process, and signal transduction (Figure 3B, Table S3). However, the KEGG pathways only included mRNA surveillance pathway and spliceosome (Table S4).

### 2.3. Comparative Parsing of LncRNAs and mRNAs and Function Analysis of DE mRNAs 

**Figure 4:**
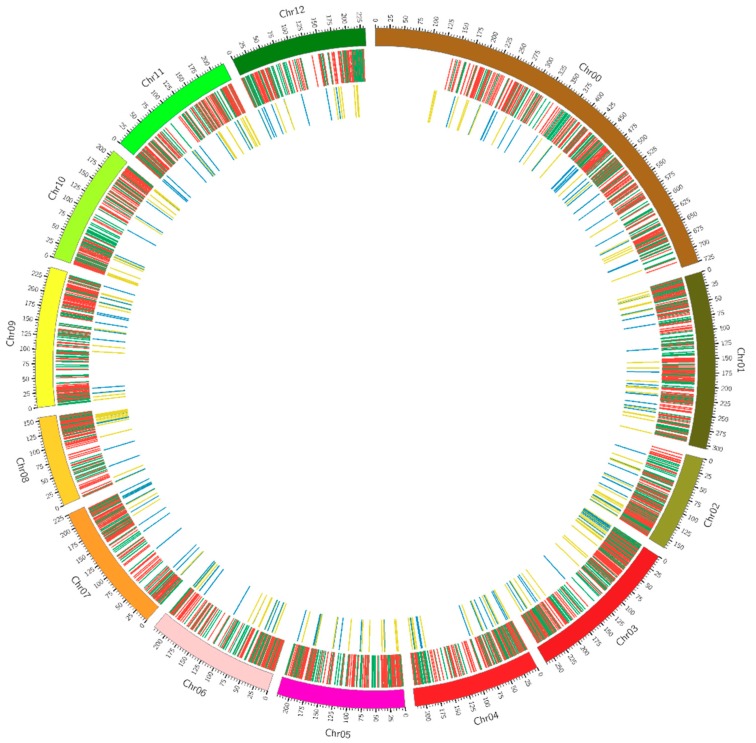
Distribution of the expression of lncRNAs and mRNAs on the different chromosomes.

There are many differences between lncRNAs and mRNAs, including their lengths, exon numbers, open reading frames, and expression levels. The number of lncRNAs decreased with the increase of the length (<3000 nt), whereas, the length of mRNAs was distributed from 400 to ≥3000 nt and had two peaks at 400 nt and ≥3000 nt. The number of the corresponding exons of lncRNAs was much less than that of mRNAs and mainly below 10; the mRNAs contained many exons, from 1 to > 30. The length of the corresponding open reading frames of lncRNAs was mainly between 50 and 300 nt, while the length of the corresponding open reading frames of mRNAs was mainly between 100 and 1100 nt (Table S5). An interactive analysis of the expression of lncRNAs and mRNAs was also conducted, and their distribution on the different chromosomes was described (Figure 4). 

For differentially expressed mRNAs, the most relevant GO terms associated with biological processes were defense response signaling pathway, response to auxin, response to abiotic stimulus, response to cold, and so on. However, the most relevant GO terms associated with molecular functions were protein kinase activity, transmembrane receptor protein kinase activity, protein serine/threonine kinase activity, signal transducer activity, and so on. KEGG pathway analysis indicated that the most frequently predicted pathways were involved in plant hormone signal transduction, phenylalanine metabolism, carbon metabolism, galactose metabolism, and so on. We found that many differentially expressed mRNAs were involved in chilling-related processes and could be divided into four different groups. The first group was transcription factors, including WRKY, MYB, bHLH, ERF, and NAC transcription factors, the second group was enzymes involved in bio-oxidation and oxidative phosphorylation, such as serine/threonine-protein kinase, polyphenol oxidase, catalase, peroxidase, lipoxygenase, and ATPase, the third group was cell wall metabolism, such as β-galactosidase, cellulose synthase, chitinase, pectate lyase, pectinesterase, and polygalacturonase, the fourth group was plant hormone-related processes, such as ethylene synthesis-related 1-aminocyclopropane-1-carboxlic acid synthase (ACS) and 1-aminocyclopropane-1-carboxlic acid oxygenase (ACO), abscisic acid receptor, gibberellin 2-β-dioxygenase, IAA-amino acid hydrolase, and salicylic acid-binding protein (Table S6).

### 2.4. Construction of the Competing Endogenous RNAs (ceRNAs) Network 

**Figure 5:**
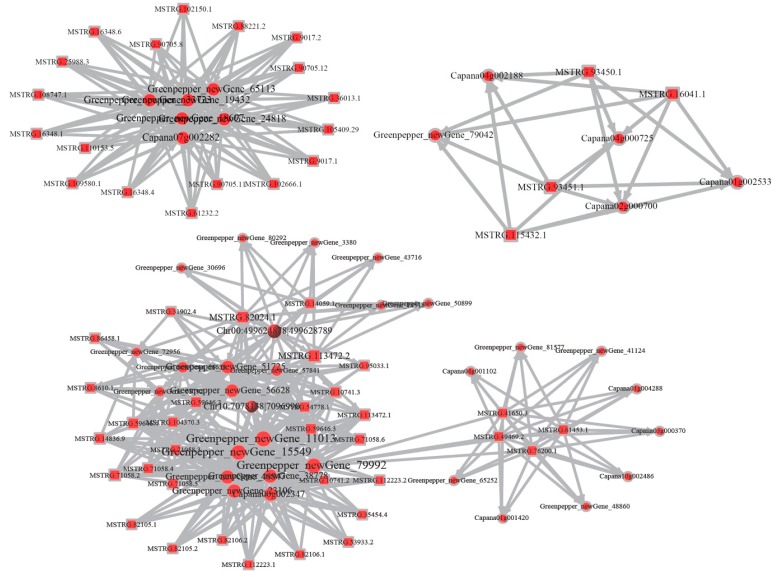
The ceRNA networks of mRNAs, lncRNAs, and circRNAs were parsed, and ceRNA relationship pairs were obtained, including 162 mRNAs, 81 lncRNAs, and 4 circRNAs.

**Figure 6:**
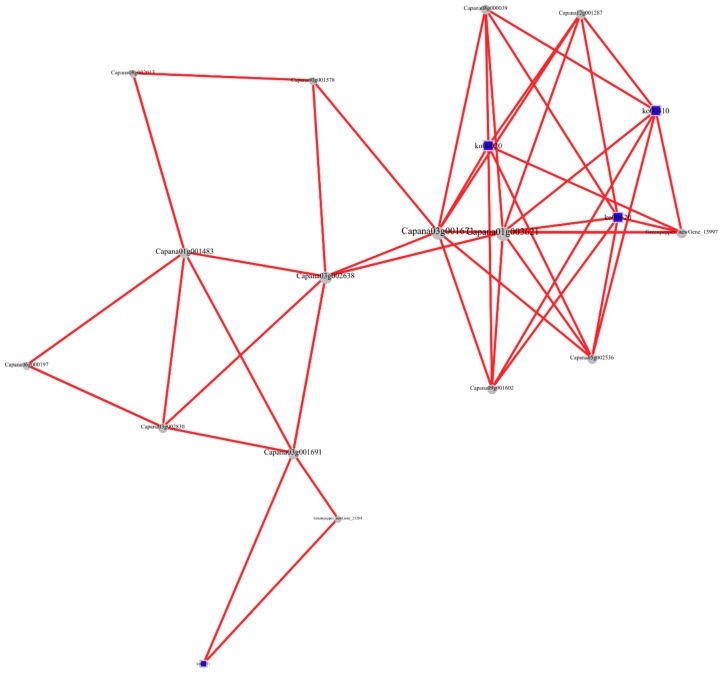
Pathway network from the annotation of the key genes. The key genes in the pathway are involved in lipid transport and metabolism, which play an important role in the chilling stress process.

It is reported that both lncRNAs and circRNAs can interact with miRNAs through microRNA response elements (MREs) within the ceRNA network [32,33]. We developed candidate ceRNA relationships through the miRNA target relationship and obtained 2972 pairs of ceRNA relationships. Then, we extracted three comprehensive ceRNA networks from the ceRNA relationship pairs, including 162 mRNAs, 81 lncRNAs, and 4 circRNAs (Figure 5, Table S7). More importantly, several important enzymes and transcription factors involved in chilling injury, such as ATPase, serine/threonine protein kinase, β-galactosidase, heat shock protein, ethylene-responsive transcription factor, were found in the ceRNA network in our results, indicating their specific cooperative regulation roles in chilling stress (Table S7). In addition, the functions of the key genes were annotated, and the first few most significantly enriched pathways were selected to extract the relationships between genes in multiple pathways and to integrate them into a pathway network. In the network, the key genes in the pathway are involved in lipid transport and metabolism which is important in the chilling stress process (Figure 6, Table S8).

## 3. Discussion

Emerging evidence shows that ncRNAs play important roles in cellular functions and especially in biotic and abiotic stresses [12]. Among all the ncRNAs, miRNAs, which perform their functions by mRNA slicing or inhibition at the post-transcriptional level, were most intensively studied [34,35]. Unlike miRNAs, the regulatory function of lncRNAs is difficult to understand because of its complexity, since lncRNAs can fold into secondary or higher orders of structure that make them more flexible in targeting proteins or gene sites [36]. Although thousands of circRNAs have been identified, their functions are largely unknown, but their spatio-temporal expression and tissue specificity indicate their potential biological roles in plants [37,38]. The cross-talk among mRNAs, lncRNA, and circRNA mediated by MREs, regulates biological processes and produces mass regulatory networks [35]. To explore the regulatory functions and complex interactions of ncRNAs in chilling injury, deep sequencing and bioinformatics technology were employed. In total, 380 lncRNAs, 36 circRNAs, 18 miRNAs, and 4128 differentially expressed mRNAs were identified, and three comprehensive ceRNA networks were found, which indicated their specific regulatory roles in chilling injury in bell pepper.

The study of ncRNAs in bell pepper is presently scanty. Few studies were focused on the regulation of lncRNAs and miRNAs in fruit development and quality in hot and black pepper [26,28,39]. At the present time, studies of ncRNA regulation in chilling injury in bell pepper are limited to the field of mRNAs [29]. This is the first report on the differential expression of lncRNA, mRNA, circRNA, and miRNA in chilling injury in bell pepper. In addition, we finely identified 9764 novel lncRNAs, 213 novel circRNAs, and 161 novel miRNAs which were enriched the ncRNAs library. Furthermore, 380 differentially expressed lncRNAs, 36 circRNAs, 18 miRNAs, and 4128 mRNAs were identified between the control and the chilling groups, which indicated their specific regulatory roles played in chilling injury. 

In order to explore the potential regulatory functions of the ncRNAs differentially expressed between control and chilling injury groups, GO analysis was performed to further annotate the biological functions of the differentially expressed ncRNAs and their target genes. We noticed that a significant amount of GO terms of the differentially expressed ncRNAs genes was related to response to abiotic stimulus, signal transduction, hormone-mediated signaling pathway, and response to cold, and the molecular functions included protein kinase activity, ATPase, and protein serine/threonine kinase activity. This phenomenon is very intriguing, revealing the vital roles that ncRNAs play in chilling injury. In accordance with the results of the GO analysis, KEGG pathway analysis also revealed pathways related to RNA degradation, peroxisome, plant hormone signal transduction, and carbon metabolism, which indicated their specific functions in the chilling response. In addition, for the differentially expressed mRNAs, numerous mRNAs which encode key enzymes, including superoxide dismutase (SOD), polyphenol oxidase (PPO), and peroxidase (POD,) involved in the protection again oxidative damage by reactive oxygen species (ROS), were found in our results. SOD converts superoxide anion (O_2_^−^) to hydrogen peroxide (H_2_O_2_), which in turn is converted to water by Catalase (CAT) and POD [40]. In our results, *SOD*, *PPO*, and *POD* were significantly upregulated, consistently with previous results [5]. Furthermore, in this study, numerous transcription factors, such as the *ERFs*, *MYB*, *NAC*, and *WRKY*, were significantly upregulated by chilling stress, which was consistent with previous results [5]. 

Recently, circRNAs were proposed to harbor miRNAs and were discovered to be enriched with functional miRNA-binding sites [41]. So far, there has been no report on ceRNAs in bell pepper fruit. Here, we constructed a lncRNA–circRNA–mRNA ceRNA network for bell pepper chilling stress based on our deep-sequencing data for the first time. In total, 162 mRNAs, 81 lncRNAs and 4 circRNAs were included in the ceRNA network. Several targets of the non-coding RNAs in the network were key enzymes in chilling injury, such as ATPase, which is an important enzyme in energy metabolism in bell pepper [42], serine/threonine protein kinase, and β-galactosidase, which are important in signaling and plant defense reaction and cell wall metabolism, respectively [37,43]. In addition, several transcription factors, such as ethylene-responsive transcription factor and heat shock factors, which play specific regulatory roles in the chilling response, were identified [44,45]. In addition, a pathway network was also constructed with the key genes of the KEGG analysis, revealing that the most important pathway was involved in lipid transport and metabolism, which are important in the chilling stress process [46,47]. These findings provide a theoretical basis for deciphering novel mechanisms of chilling injury and for the functional characterization of ceRNA networks in the future studies.

## 4. Materials and Methods

### 4.1. Sample Collection and Preparation

Green bell peppers (*C. annuum* L. cv. Jingtian) were harvested from a green house in the “Xiaotangshan” and quickly transported to the lab. The control fruits were stored at 10 °C, whereas the chilled fruits were stored at 1 °C for 72 h. Bell pepper fruit pericarp samples were collected, frozen in liquid nitrogen, and stored at −80 °C for the subsequent experiments.

### 4.2. Methods of RNA Extraction and Detection

The RNA samples were extracted with RNA Extraction Kit (RN40, Aidlab Biotechnologies, Beijing, China). RNA integrity was assessed using the RNA Nano 6000 Assay Kit of the Agilent Bioanalyzer 2100 system (Agilent Technologies, Santa Clara, CA, USA), to ensure the use of qualified samples for sequencing.

Library preparation for sRNA sequencing: A total amount of 2.5 ng RNA per sample was used as input material for the RNA sample preparations. Sequencing libraries were generated using NEB Next Ultra small RNA Sample Library Prep Kit for Illumina (NEB, Ipswich, MA, USA), following the manufacturer’s recommendations, and index codes were added to attribute sequences to each sample. First of all, the 3′SR Adaptor was ligated and mixed for Illumina. The RNA and nuclease-free water were mixed after incubation for 2 min at 70 °C in a preheated thermal cycler, which was then transferred to ice. A 3′Ligation Reaction Buffer (2×) was then added and mixed with the 3′Ligation Enzyme Mix, after which, the 3′SR Adaptor was ligated and incubated for 1 h at 25 °C in a thermal cycler. To prevent adaptor–dimer formation, the SR RT Primer hybridizes to the excess of 3′SR Adaptor (that remains free after the 3′ligation reaction) and transforms the single-stranded DNA adaptor into a double-stranded DNA molecule (dsDNAs) that is not a substrate for ligation. Subsequently, the 5′SR Adaptor was ligated. Then, reverse transcription produced the synthetic first chain. Last, PCR amplification and Size Selection were performed. A polyacrylamide gel electrophoresis (PAGE) gel was used for fragment screening, rubber cutting recycling as the pieces get small RNA libraries. At last, the PCR products were purified (AMPure XP system, Beckman Coulter, Beverly, MA, USA), and the library quality was assessed on the Agilent Bioanalyzer 2100 system (Agilent Technologies, Santa Clara, CA, USA). 

Library preparation for lncRNAs and circRNAs sequencing: A total amount of 1.5 μg RNA (for circRNA it was 2.0 μg) per sample was used as input material for rRNA removal using the Ribo-Zero rRNA Removal Kit (Epicentre, Madison, WI, USA). Sequencing libraries were generated using NEBNext^®^ Ultra™ Directional RNA Library Prep Kit for Illumina^®^ (NEB, USA), following the manufacturer’s recommendations, and index codes were added to attribute sequences to each sample. Briefly, fragmentation was carried out using divalent cations under an elevated temperature in NEBNext First-Strand Synthesis Reaction Buffer (5×). First-strand cDNA was synthesized using random hexamer primers and Reverse Transcriptase. Second-strand cDNA synthesis was subsequently performed using DNA Polymerase I and RNase H. The remaining overhangs were converted into blunt ends via exonuclease and polymerase activities. After adenylation of the 3’ ends of the DNA fragments, NEBNext Adaptor with a hairpin loop structure was ligated to prepare for hybridization. In order to select insert fragments of preferentially 150–200 bp (for circRNA it was 150–250 bp) in length, the library fragments were purified with AMPure XP Beads (Beckman Coulter). Then, 3 μL USER Enzyme (NEB, USA) was used with size-selected, adaptor-ligated cDNA at 37 °C for 15 min before PCR. Then PCR was performed with Phusion High-Fidelity DNA polymerase, Universal PCR primers and Index(X) Primer. At last, the PCR products were purified (AMPure XP system), and the library quality was assessed on the Agilent Bioanalyzer 2100 and qPCR.

### 4.3. Clustering, Sequencing, and Quality Control

The clustering of the index-coded samples was performed on a cBot Cluster Generation System using TruSeq PE Cluster Kit v4-cBot-HS (Illumina) according to the manufacturer’s instructions. After cluster generation, the library preparations were sequenced on an Illumina Hiseq platform, and paired-end reads were generated. The raw data (raw reads) of fastq format were firstly processed through in-house perl scripts. In this step, clean data (clean reads) were obtained by removing reads containing adapters, reads containing ploy-N, and low-quality reads from the raw data. At the same time, Q20, Q30, GC content, and sequence duplication level of the clean data were calculated. All the downstream analyses were based on clean data with high quality (Supplied by BioMarker, Beijing, China).

### 4.4. NcRNAs Identity

The transcriptome was assembled using the StringTie (https://ccb.jhu.edu/software/stringtie/index.shtml) [48] based on the reads mapped to the reference genome. The assembled transcripts were annotated using the gff compare program (Cuffcompare 2.2.1, http://cole-trapnell-lab.github.io/cufflinks/manual/). The unknown transcripts were used to screen for putative lncRNAs. Three computational approaches, namely, CPC (0.9-r2, http://cpc.cbi.pku.edu.cn/)/CNCI(v2, http://www.ncbi.nlm.nih.gov/pubmed/23892401)/Pfam(v1.5, http://pfam.xfam.org/)/CPAT(v1.2.2, http://lilab.research.bcm.edu/cpat/) [49,50,51,52], were combined to sort non-protein-coding RNA candidates from putative protein-coding RNAs in the unknown transcripts. Putative protein-coding RNAs were filtered out using a minimum length and exon number threshold. Transcripts with lengths over 200 nt and with more than two exons were selected as lncRNA candidates and further screened using CPC/CNCI/Pfam/CPAT that have the power to distinguish protein-coding genes from non-coding genes. The different types of lncRNAs, including long intergenic noncoding RNAs (lincRNAs), intronic lncRNAs, anti-sense lncRNAs, sense lncRNAs were selected using cuff compare (Cuffcompare 2.2.1, http://cole-trapnell-lab.github.io/cufflinks/manual/)(Supplied by BioMarker).

We used CIRI (CircRNA Identifier, v2.0.5) [53] tools to identify circRNA; it scans SAM files twice and collects sufficient information to identify and characterize circRNAs. Briefly, during the first scanning of SAM alignment, CIRI detects junction reads with PCC signals that reflect a circRNA candidate. Preliminary filtering is implemented using paired-end mapping (PEM) and GT–AG splicing signals for the junctions. After clustering the junction reads and recording each circRNA candidate, CIRI scans the SAM alignment again to detect additional junction reads and, meanwhile, performs further filtering to eliminate false-positive candidates resulting from incorrectly mapped reads of homologous genes or repetitive sequences. Finally, the identified circRNAs are output with annotation information.

Using Bowtie software, the clean reads were analyzed respectively with Silva database, GtRNAdb database, Rfam database, and Repbase database sequence alignment, to filter ribosomal RNA (rRNA), transfer RNA (tRNA), small nuclear RNA (snRNA), small nucleolar RNA (snoRNA), and other ncRNA and repeats. The remaining reads were used to detect known miRNA and novel miRNA, predicted by comparing with known miRNAs from the miRBase. Randfold tools soft (v2.1.7) was used for novel miRNA secondary structure prediction (Supplied by BioMarker, Beijing, China).

### 4.5. Differential Expression Analysis

Differential expression analysis of two conditions or groups was performed using the DESeq R package (1.18.0, http://www.bioconductor.org/packages/release/bioc/html/DESeq.html) [54]. DESeq provides statistical routines for determining differential expression in digital gene expression, lncRNAs, circRNAs, and miRNAs expression data, using a model based on the negative binomial distribution. The resulting P values were adjusted using the Benjamini and Hochberg’s approach for controlling the false discovery rate. Genes, lncRNAs, and circRNAs with an adjusted *p*-value < 0.01 and an absolute value of log2 (Fold change) > 1 found by DESeq were assigned as differentially expressed. miRNAs with an adjusted *p* < 0.05 found by DESeq were assigned as differentially expressed (Supplied by BioMarker, Beijing, China).

### 4.6. Gene Function Annotation

Gene function was annotated on the basis of the following databases: Nr (NCBI non-redundant protein sequences; ftp://ftp.ncbi.nih.gov/blast/db/FASTA/); Pfam (Protein family; http://pfam.xfam.org/); KOG/COG (Clusters of Orthologous Groups of proteins; http://www.ncbi.nlm.nih.gov/KOG); Swiss-Prot (A manually annotated and reviewed protein sequence database; http://www.uniprot.org/); KEGG (Kyoto Encyclopedia of Genes and Genomes; http://www.genome.jp/kegg/); GO (Gene Ontology; http://www.geneontology.org/). 

### 4.7. GO and KEGG Pathway Enrichment Analysis 

GO enrichment analysis of the differentially expressed genes (DEGs) was implemented by the GOseq R packages based on Wallenius non-central hyper-geometric distribution. We used KOBAS software to test the statistical enrichment of differentially expressed genes in KEGG pathways [55].

### 4.8. CeRNAs Network Analysis of ncRNAs

A hypergeometric test was executed for each ceRNA pair separately, which was defined by four parameters: (i) N was the total number of miRNAs used to predict targets; (ii) K was the number of miRNAs that interact with the chosen gene of interest; (iii) n was the number of miRNAs that interact with the candidate ceRNA of the chosen gene; (iv) c was the common miRNA number between these two genes. The test calculates the P-value by using the following formula:

$$\;P=\overset{\min\left(K,n\right)}{\underset{i=c}{\sum }}\frac{\left(\begin{array}{c} K\\ i \end{array}\right)\left(\begin{array}{c} N-K\\ n-i \end{array}\right)}{\left(\begin{array}{c} N\\ n \end{array}\right)}$$

All *p*-values were subject to false discovery rate (FDR) correction. The following features were necessary for ceRNAs: (i) number of miRNAs that interact with the candidate ceRNA ≥ 5; (ii) FDR < 0.05 [56].

## Supplementary Materials

Supplementary materials can be found at http://www.mdpi.com/1422-0067/19/7/2001/s1.
